# Higher internal locus of control is associated with higher performance in a workplace walking intervention, Global Corporate Challenge®

**DOI:** 10.1371/journal.pone.0349934

**Published:** 2026-06-01

**Authors:** Haoxiong Sun, Pei-Chun Ko, S. Fiona Barker, Danijela Gasevic, Jessica Stone, Rosanne Freak-Poli

**Affiliations:** 1 School of Clinical Sciences at Monash Health, Monash University, Melbourne, VIC, Australia; 2 Department of Behavioural Science and Health, University College London, London, United Kingdom; 3 School of Social Sciences, Monash University, Melbourne, VIC, Australia; 4 School of Public Health and Preventive Medicine, Monash University, Melbourne, VIC, Australia; 5 Centre for Global Health, Usher Institute, The University of Edinburgh, Edinburgh, United Kingdom; ISSEP Kef: Universite de Jendouba Institut Superieur du Sport et de l'Education Physique du Kef, TUNISIA

## Abstract

**Background:**

Given the increasing reliance on self-regulation in modern work environments, understanding how psychological characteristics influence physical activity participation can help inform more effective health promotion strategies. This study examines whether individuals with different Locus of Control (LOC) orientations show varying levels of engagement and performance in a workplace pedometer program.

**Methods:**

We conducted a secondary analysis of 426 office workers in Melbourne, Australia who participated in the 2008 Global Corporate Challenge® (GCC®), a four-month team-based pedometer program encouraging 10,000 steps per day. Internal LOC (ILOC) was assessed at baseline, four months, and 12 months using the Duttweiler Internal Control Index. Baseline ILOC was the primary exposure. Program performance was operationalized as average daily step counts and achievement of the 10,000-step daily goal. Linear regression and logistic regression, adjusted for workplace clustering and covariates, were used to examine associations between ILOC and outcomes. Paired t-tests and descriptive comparisons were used to assess changes in ILOC over time.

**Results:**

Participants with higher baseline ILOC were older (p < 0.001), more likely to have previously completed the GCC® (p = 0.039), more health-motivated (p = 0.015), met fruit (p = 0.040) and vegetable (p = 0.003) intake guidelines, and reported higher wellbeing and mental health-related quality of life (both p < 0.001). During the program, higher ILOC was associated with higher average daily step counts (β = 46.97, 95% CI [24.62, 69.33]; p = 0.001) and greater likelihood of achieving the 10,000-step goal (OR = 1.02, 95% CI [1.01, 1.03]; p = 0.001). ILOC showed no significant net change overall, but a clear regression-to-the-mean pattern was observed across baseline quartiles.

**Conclusions:**

Higher ILOC was associated with better performance in the pedometer program, though this did not translate into sustained additional health gains.

## Background

In occupational health, there is an increasing dependence on individuals’ capacity for self-regulation to achieve and sustain physical activity gains [1–3]. This reliance on individual self-regulatory capacity has become more important as flexible and hybrid work arrangements reduce the external structures that traditionally supported occupational health in onsite work environments [4–7]. Consequently, understanding how internal psychological characteristics shape engagement with workplace health programs becomes increasingly important for designing interventions that function effectively in diverse work environments.

A key psychological construct that captures individual differences in self-regulatory orientation is the Locus of Control (LOC), first conceptualized by Rotter [8]. It is defined as the degree to which individuals attribute outcomes to their own actions (internal) versus external circumstances (external) [8]. Those with a higher internal locus of control (ILOC) believe their actions matter. They take responsibility for their behavior and tend to act proactively toward health goals. Self-Determination Theory (SDT) adds that people with greater internal control also show more autonomous motivation, which helps them maintain healthy behavior and supports psychological wellbeing [9,10]. These highlight that ILOC may influence both whether individuals participate, and how effectively they maintain engagement in health promotion programs.

Contemporary workplace interventions increasingly rely on digital health technologies, including smartphone-based fitness trackers and challenges, and app-based gamification elements, like points or badges [11–13]. While these tools have improved accessibility and engagement, they also introduce external stimuli, like competitive leaderboards and algorithm factors, making it difficult to disentangle the psychological from the technological determinants of behavior change [12,14,15]. To isolate the influence of intrinsic psychological characteristics on health behavior, it is therefore valuable to examine data from workplace programs conducted before the widespread adoption of these digital tools. The Global Corporate Challenge® (GCC), a structured pedometer-based program conducted in Australia in 2008, thus serves as a valuable “pre-digital benchmark” for understanding the enduring psychological mechanisms underpinning workplace health engagement [16–19]. Because LOC is often treated as a relatively stable non-cognitive trait, findings from pre-digital workplace interventions remain useful for examining intrinsic drivers of behavior with minimal influence from modern digital health tools [20–22].

Previous evidence has demonstrated the effectiveness of the GCC in promoting physical activity [16–19]. Therefore, the primary objective of this study is to investigate whether baseline ILOC is associated with average daily step counts and 10,000-step goal attainment in this workplace pedometer program. Secondary outcome includes examining the distribution of ILOC among participants, and identifying patterns of ILOC change throughout the intervention.

## Methods

### Design and sample

This study is a secondary analysis of an existing, fully de-identified sample of office workers from Melbourne, Australia who were in predominantly sedentary occupations and enrolled in a group-based, pedometer workplace program.

The original Global Corporate Challenge® (GCC®) Evaluation Study was approved by the Monash University Human Research Ethics Committee through the Standing Committee on Ethics in Research involving Humans (Low Impact Research Project Involving Humans, project number CF08/0217–2008000125). Written informed consent was obtained from all participants at the time of the original study. The present study is a secondary analysis of a fully de-identified dataset from the GCC® Evaluation Study, and the authors signed the GCC® Evaluation Study Data Use Agreement. In accordance with Monash University research ethics policy, no additional ethics approval was required for this secondary analysis. The data were accessed on September 2024.

### Intervention

The Global Corporate Challenge® (GCC®) is a corporate organisation that held a world-wide workplace health program that used pedometers to track individuals’ physical activity in 2008. GCC® program participants were organised into teams of seven workers and were required to meet an individual step goal of 10,000 steps per day, the recommended step goal at the time the program was run [23]. Participants were asked to wear the pedometers provided by the GCC® all the time with the exception of swimming, showering, and sleeping. The team’s progress (i.e., steps travelled) was displayed virtually as moving around a world map, with information on locations as they arrived. Participants were able to compare their team’s progress to other teams in their company worldwide. Participants were sent weekly encouragement newsletters via email including the participant’s personal best daily step count, health tips from a nutritionist, stories from other participants, a “Dear GCC” section answering participants’ questions, housekeeping and prizes awarded by sponsors of the program. A website was used for logging daily step counts and provided access to additional health information such as the number of steps required to burn off a hamburger, communication among participants and comparing team progress.

### Sample

**Fig 1:**
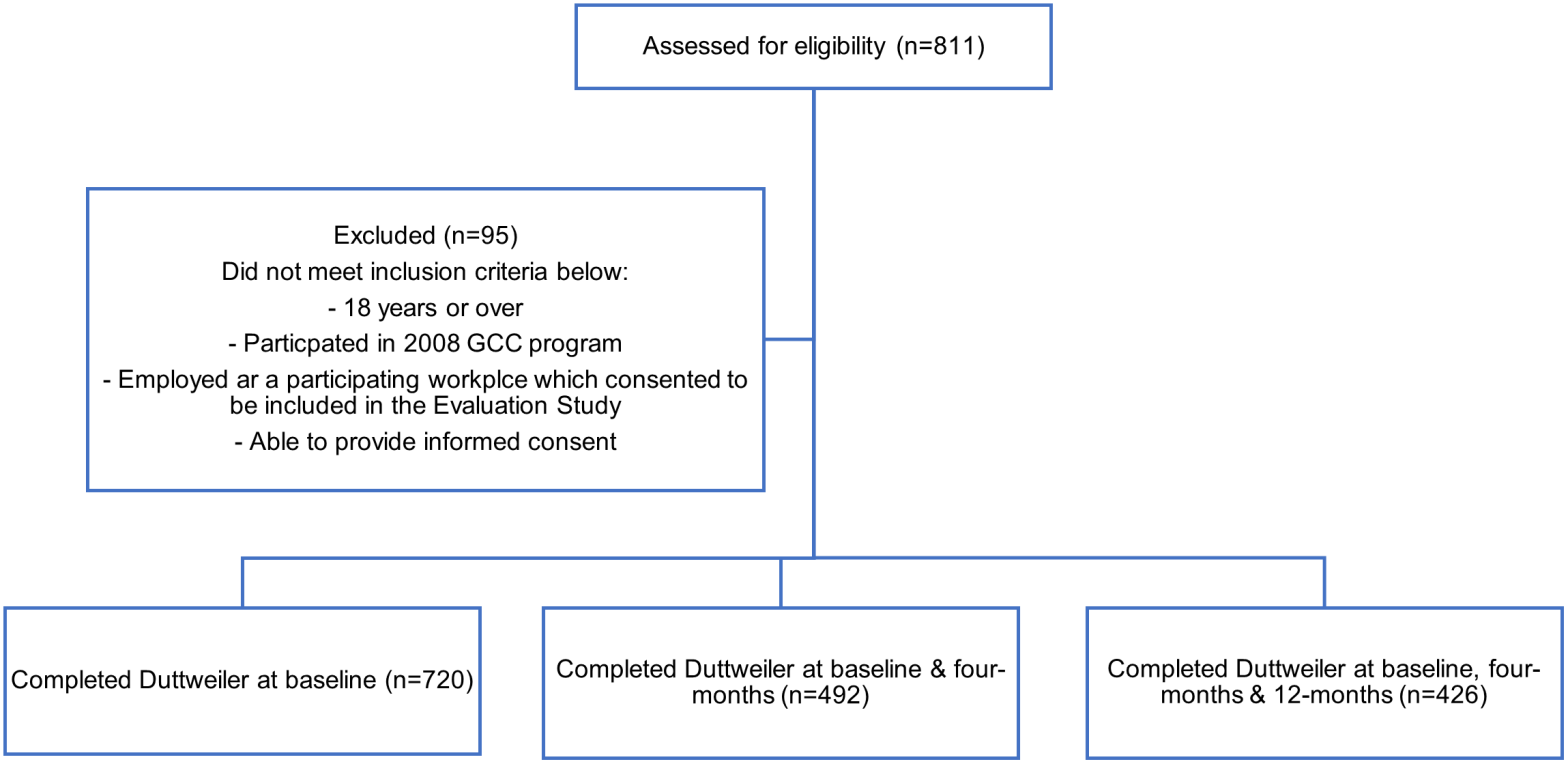
Participant recruitment.

The GCC® Evaluation Study was a prospective cohort study conducted over a 12-month period in 2008 in workplaces across Melbourne. Participants were recruited from ten, predominantly sedentary workplaces, over eight weeks in April and May 2008. The 811 participants enrolled in the Evaluation Study were also participants of the GCC® program, and this study focused on the 426 participants who completed the LOC at baseline, four- and 12-month follow-up data collection (Fig 1). To address potential selection bias due to attrition, we compared baseline characteristics between the analytic sample (n = 426) and those excluded or lost to follow-up (n = 385). While retained participants were older (p < 0.001), there were no other significant differences, and age was included as a covariate in all models to adjust for the demographic difference (S1 Table).

### Measures

#### Exposure: Internal Locus of Control (ILOC).

ILOC can be measured using a variety of questionnaires, which can be dominated by internal- or external-focused questions [24]. We assessed ILOC using the Duttweiler Internal Control Index [25], which is considered a general ILOC scale with good reliability (alpha = 0.85) and exhibits concurrent validity compared to other general scales [24]. The Duttweiler Internal Control Index is a 28-item questionnaire with responses in a 5-point scale ranging from (A) “rarely” to (E) “usually” equating to a score ranging from 28 (low internal control) to 140 (high internal control). Negatively worded items were reverse-coded so that higher scores indicated higher internal control. In this study, ILOC was treated as both a continuous and a categorical variable, depending on the analysis. When treated as a categorical variable, ILOC scores were divided into quartiles based on the baseline distribution of the analytic sample: very low (65–98), low (99–107), high (108–113), and very high (114–136)

#### Outcome.

Daily step counts were collected using GCC® brand pedometers (internally validated). Step data were entered daily by participants and averaged across all days with recorded entries during the 16-week program. The GCC® platform required manual daily entry, and days without entries were excluded from the denominator. As the GCC® platform provided immediate feedback to participants during data entry, no post-hoc outlier exclusion criteria were applied to the averaged data. The program encouraged a daily step goal of 10,000 steps based on evidence from Tudor-Locke that suggests 10,000 daily steps are indicative of active individuals [26]. Behavioral measures were collected using the WHO STEPwise approach (WHO, 2002). Participants were classified as ‘meeting physical activity guidelines’ (Yes/No) if they reported achieving at least 150 minutes of moderate-intensity physical activity per week, in accordance with WHO recommendations.

#### Covariates.

Covariates were selected based on a prior GCC®-related study investigating mental health issues [16,27,28]. A detailed list of covariates and their subtypes is provided (S5 Table). Demographic information (age, sex, tertiary education, partner status, socio economic status, occupation), prior participation in the GCC®, motivation for participation (health, to look my best, fitness, colleagues or friends and family) and behavioural measures (fruit and vegetable intake, alcohol intake, smoking status, physical activity, sitting time and takeaway dinner consumption) were collected using the core and expanded options of the WHO STEPwise approach [29] and the WHO mini-STEP [30]. Psychosocial measures of wellbeing were collected using the WHO-5 questionnaire, health-related quality of life was measured using the SF-12, and psychological distress (Kessler Psychological Distress Scale, K10) [27].

Anthropometric measures including blood pressure, heart rate, weight, body mass index (BMI), and waist circumference were measured at baseline, four-months and twelve-months. Measurements were conducted by trained staff in the morning at the employee’s workplaces using the following equipment: blood pressure (Omron IA1B Automatic blood pressure IntelliSense machine), height (stadiometer portable height scale code PE087 and step ladder), weight (Salter electronic bathroom scales model 913 WH3R 3007 during baseline and four-month data collection, and Seca digital scales model Robusta 813 during twelve-month data collection). Waist circumference was measured at the midpoint between the lower margin of the last palpable rib and the top of the iliac crest following WHO guidelines (Figure Finder Tape Measure Novel Products Inc 2005 code PE024 and a mirror) [28].

#### Analysis.

All statistical analyses were performed using Stata 16.0 (StataCorp, College Station, TX). To account for the clustered nature of the data (employees nested within workplaces), survey estimation commands (svy) were used for all regression analyses, with “workplace” defined as the primary sampling unit.

Attrition analysis was conducted to compare baseline characteristics between participants included in the final analysis and those excluded or lost to follow-up, using independent t-tests for continuous variables and chi-square tests for categorical variables.

Baseline participant characteristics were summarized by quartiles of ILOC level using mean (SD) for continuous variables and counts (percentages) for categorical variables. Differences across the four LOC quartiles were evaluated using linear regression for continuous variables and logistic regression for categorical variables to test for linear trends.

To assess the association between baseline LOC and outcomes, two types of regression models were used. Linear regression analyses (svy: reg) were conducted for continuous outcomes (e.g., average daily steps, changes in well-being, K10 scores, and anthropometric measures). The unstandardized regression coefficient (β) represents the mean change in the outcome for each 1-unit increase in LOC. Logistic regression analyses (svy: logit) were used for binary outcomes (e.g., meeting the 10,000-step goal, meeting physical activity guidelines), with results reported as Odds Ratios (ORs) and 95% confidence intervals.

Multiple regression models were fitted to test the robustness of the findings after adjusting for demographic (age, sex), behavioral, and health-related covariates (BMI, baseline values). Paired t-tests were used to evaluate the mean change in LOC scores from baseline to the 4- and 12-month follow-ups. Statistical significance was set at p < 0.05 for all tests.

## Results

### ILOC, baseline covariates, and attrition analysis

Participants with higher ILOC were, on average, older (p < 0.001), more likely to have previously participated in the GCC® (p = 0.039), and more likely to report health-related motivations for participating (p = 0.015). Regarding health behaviors at baseline, those with higher ILOC were more likely to meet guidelines for fruit intake (p = 0.040) and vegetable intake (p = 0.003). Psychologically, higher ILOC was associated with significantly higher well-being scores (p < 0.001), higher mental health-related quality of life (SF-12 MCS, p < 0.001), and lower psychological distress (K10, p < 0.001) at baseline (Table 1).

**Table 1:** Baseline characteristics of participants with very low, low, high and very high internal locus of control (Duttweiler scores).

Locus of Control (Duttweiler) ^a^						
	All participants	Very Low ILoC	Low ILoC	High ILoC	Very High ILoC	p-value ^b^
	Mean±SD or (n)%	Mean±SD or (n)%	Mean±SD or (n)%	Mean±SD or (n)%	Mean±SD or (n)%	
**n**	426	113	110	100	103	
**DEMOGRAPHICS**						
Age (year)	41.3 ± 10.2	38.3 ± 9.9	40.9 ± 10.6	43.4 ± 9.8	43.1 ± 9.8	**<0.001**
Male	(180)42.25%	(49)43.4%	(54)49.1%	(35)35%	(42)40.8%	0.227
Completion of tertiary education ^c^	(344)80.75%	(88)77.9%	(87)79.1%	(85)85%	(84)81.6%	0.225
Partner status						
Married or de facto	(302)70.89%	(77)68.1%	(82)74.6%	(72)72%	(71)68.9%	0.781
Widowed, separated or divorced	(42)9.86%	(11)9.7%	(10)9.1%	(9)9%	(12)11.7%	
Never married	(82)19.25%	(25)22.1%	(18)16.4%	(19)19%	(20)19.4%	
Socio Economic Status (by SEIFA)						
Most Advantaged	(101)23.76%	(28)24.8%	(22)20%	(27)27%	(24)23.5%	0.478
Advantaged	(113)26.59%	(31)27.4%	(33)30%	(25)25%	(24)23.5%	
Disadvantaged	(109)25.65%	(33)29.2%	(25)22.7%	(20)20%	(31)30.4%	
Most Disadvantaged	(102)24.00%	(21)18.6%	(30)27.3%	(28)28%	(23)22.6%	
Occupation						
Professional	(189)47.61%	(46)46.9%	(55)51.4%	(46)48.4%	(42)43.3%	0.758
Associate professional	(78)19.65%	(20)20.4%	(19)17.8%	(18)19%	(21)21.7%	
Manager	(76)19.14%	(12)12.2%	(17)15.9%	(22)23.2%	(25)25.8%	
Clerical or Service	(54)13.6%	(20)20.4%	(16)15%	(9)9.5%	(9)9.3%	
**GCC Intervention measures**						
Prior GCC® Participation^c^	(98) 23%	(17)15%	(29)26.4%	(28)28%	(24)23.3%	**0.039**
Motivation for participation						
Health^c^	(292) 68.54%	(89)78.8%	(69)62.7%	(70)70%	(64)62.1%	**0.015**
To look my best^c^	(261) 61.27%	(72)63.7%	(67)60.9%	(66)66%	(56)54.4%	0.22
Fitness^c^	(288) 67.61%	(77)68.1%	(68)61.8%	(75)75%	(68)66%	0.685
Colleagues^c^	(416)97.65%	(110)97.4%	(107)97.3%	(97)97%	(102)99%	0.095
Friends or family^c^	(51) 11.97%	(13)11.5%	(15)13.6%	(12) 12.00%	(11)10.7%	0.814
**Behavioural measures**						
Fruit intake (meeting guidelines)^c^	(142) 33.33%	(33)29.2%	(28)25.5%	(42)42%	(39)37.9%	**0.04**
Vegetable intake (meeting guidelines)^c^	(67) 15.73%	(6)5.3%	(19)17.3%	(19)19%	(23)22.3%	**0.003**
Alcohol (meeting guidelines)^c^	(181) 42.49%	(47)41.6%	(53)48.2%	(36)36%	(45)43.7%	0.853
Non smoker^c^	(393) 92.25%	(108)95.6%	(98)89.1%	(92)92%	(95)92.2%	0.377
Physical activity (meeting guidelines)^c^	(172) 41.55%	(47)41.6%	(40)36.4%	(42)42%	(48)46.6%	0.081
Sitting time (hrs per day)						
Weekday	8.27 ± 3.6	7.6 ± 3.8	9 ± 3.7	8.2 ± 3.5	8.3 ± 3.3	0.568
Weekend	5.33 ± 2.87	5.1 ± 2.7	5.8 ± 2.9	5.5 ± 3.1	5 ± 2.8	0.753
Takeaway Dinner						
Once or less per month	(199) 46.71%	(44)38.9%	(49)44.6%	(53)53%	(53)51.5%	0.1
About once a week	(176) 41.31%	(54)47.8%	(46)41.8%	(38)38%	(38)36.9%	
More than once a week	(51) 11.97%	(15)13.3%	(15)13.6%	(9)9%	(12)11.7%	
**Psychosocial measures**						
Well-being	(424)60.08 ± 18.92	(112)51.4 ± 20.6	(110) 57.3 ± 17.6	(100) 63.8 ± 16.8	(102)68.9 ± 15.4	**<0.001**
Well-beingc (positive category)	(318) 75%	(64)57.1%	(82)74.6%	(78)78%	(94)92.2%	**<0.001**
Health related quality of life (SF-12)						
Mental health component	(422)49.36 ± 9.93	(112)45.5 ± 11.3	(109)49.1 ± 9.4	(100) 51.1 ± 7.6	(101)52.1 ± 9.7	**<0.001**
Physical health component	(422)50.949 ± 7.45	(112)50.1 ± 8.2	(109)51.3 ± 7.1	(100)51.4 ± 6.9	(101)51.1 ± 7.5	0.274
K10 Scores						
Low	(180) 42.45%	(16)14.3%	(26)23.6%	(30)30%	(45)44.1%	**<0.001**
Moderate	(153) 36.08%	(51)45.5%	(61)55.5%	(60)60%	(44)43.1%	
High	(72) 16.98%	(37)33%	(17)15.5%	(8)8%	(3)2.9%	
Very high	(19) 4.48%	(8)7.1%	(6)5.5%	(2)2%	(3)2.9%	
**Anthropometric measures**						
Systolic blood pressure (mmHg)	118.8 ± 14.2	(106)117.3 ± 14.1	(106)120.7 ± 15.7	(96)117.7 ± 12.9	(96)119.6 ± 13.7	0.689
Diastolic blood pressure (mmHg)	79.9 ± 10.2	(106)78.7 ± 9.9	(106)81.8 ± 11.1	(96)78.5 ± 9.5	(96)80.5 ± 9.7	0.75
Heart rate (beats per minute)	68.6 ± 10.1	(106)69 ± 11	(106)69 ± 9.2	(96)68.4 ± 10.2	(96)67.9 ± 10.1	0.434
Weight (kg)	77.4 ± 15.8	(108)75.6 ± 16.7	(109)79.9 ± 17.2	(97)76.9 ± 13.4	(100)77.1 ± 15.4	0.796
Body mass index (kg/m2)	26.8 ± 4.8	(108)26.2 ± 4.9	(109)27.3 ± 5.4	(97)26.9 ± 4.1	(100)26.9 ± 4.8	0.577
Waist circumference	88.4 ± 12.6	(108)87.5 ± 13.1	(109)90 ± 12.7	(97)88.2 ± 12.2	(100)87.7 ± 12.4	0.876

^a^Restricted to participants who attended and completed the Duttweiler Internal Control Index at baseline, four-month and 12-month data collection.

^b^Bold highlights statistically significant results.

^c^The reference group for this binary variable is ‘no’. The reference group data is not shown.

Attrition analysis comparing participants included in the final analysis (n = 426) with those excluded or lost to follow-up (n = 385) revealed that retained participants were significantly older (41.3 vs. 37.9 years, p < 0.001). However, no significant differences were observed in sex, education level, BMI, or baseline LOC scores between the groups (S1 Table).

### Association between ILOC and intervention performance

After adjusting for clustering and covariates (age, sex, BMI, and baseline health metrics), baseline ILOC had a significant association with physical activity outcomes. For every one-unit increase in baseline ILOC, average daily steps increased by approximately 48 steps (β = 46.97, 95% CI [24.62, 69.33]; p = 0.001), corresponding to an increase of approximately 521 steps per standard deviation (SD = 11.1) in ILOC (S3 Table).

Similarly, logistic regression analysis indicated that higher ILOC was associated with a greater likelihood of achieving the 10,000-step daily goal (OR = 1.02, 95% CI [1.01, 1.03]; p = 0.001). Furthermore, contrary to unadjusted observations, after full adjustment, baseline ILOC was not significantly associated with meeting WHO physical activity guidelines at four months (OR = 1.01, 95% CI [1.00, 1.03]; p = 0.115) (Table 2).

**Table 2:** Daily step counts and meeting 10,000-step goal by ILOC.

Outcome	Descriptive Statistics by ILOC Quartile				Association with ILOC score^a^	
	Very Low Mean±SD	Low Mean±SD	High Mean±SD	Very High Mean±SD	Coefficient ^b^	P-value
**Daily Steps**	11,218 ± 3,573	11,165 ± 3,998	12,373 ± 3,313	12,441 ± 3,904	46.97 (24.62, 69.33)	0.001
**Meeting 10,000 Step Goal**	**Very Low** n (%)	**Low** n (%)	**High** n (%)	**Very High** n (%)	**OR (95% CI)** ^c^	**P-value**
Yes	72 (63.7%)	65 (59.6%)	80 (80.0%)	74 (71.8%)	1.022 (1.012, 1.032)	0.001
No	41 (36.3%)	44 (40.4%)	20 (20.0%)	29 (28.2%)	Reference	
**Meeting PA Guidelines (at 4 months)**	**n (%)**	**n (%)**	**n (%)**	**n (%)**	**OR (95% CI)**	**P-value**
Yes	46 (40.7%)	46 (41.8%)	55 (55.0%)	59 (57.3%)	1.014 (0.996, 1.032)	0.115
No	67 (59.3%)	64 (58.2%)	45 (45.0%)	44 (42.7%)	Reference	

Q1-Q4 represents quartiles of baseline ILOC scores.

^a^Results from fully adjusted models (Model 3) accounting for workplace clustering and adjusted for age, sex, BMI, baseline physical activity, sitting time, well-being, mental/physical health, blood pressure, and waist circumference.

^b^Value represents unstandardized linear regression coefficient (increase in steps per 1-unit ILOC increase).

^c^Value represents Odds Ratio (OR) from logistic regression (likelihood of meeting goal per 1-unit ILOC increase).

### Association between internal LOC and health outcomes

Longitudinal associations between baseline ILOC and health outcomes were examined at 4 and 12 months (Table 3). Regarding mental health outcomes, higher baseline ILOC was associated with better mental health-related quality of life (SF-12 MCS) at both 4 months (β = 0.10, p = 0.010) and 12 months (β = 0.09, p = 0.001). Interestingly, while overall mental health quality was higher, higher ILOC was also associated with increased psychological distress (K10) at 4 months (β = 0.07, p = 0.007); however, this association was no longer significant at 12 months. No significant associations were observed for well-being (WHO-5) changes at either time point.

**Table 3:** Associations between baseline ILOC and health outcomes at 4 months and 12 months.

Outcome	4-Month Follow-up		12-Month Follow-up	
Mental Health	Coeff. (95% CI)	P-value	Coeff. (95% CI)	P-value
Well-being (WHO-5)	0.11 (−0.03, 0.25)	0.104	0.16 (−0.05, 0.37)	0.116
SF-12 MCS	0.10 (0.03, 0.17)	**0.010**	0.09 (0.05, 0.13)	**0.001**
Psychological distress (K10 total score)	0.066 (0.023, 0.109)	**0.007**	0.025 (−0.012, 0.063)	0.159
**Physical Health**	**Coeff. (95% CI)**	**P-value**	**Coeff. (95% CI)**	**P-value**
SF-12 PCS	0.04 (0.01, 0.07)	**0.022**	0.02 (−0.03, 0.08)	0.325

Values represent unstandardized linear regression coefficients (β) and 95% Confidence Intervals (CI). Analyses were adjusted for workplace clustering and controlled for baseline scores of the respective outcome, age, sex, and BMI. Abbreviations: WHO-5, World Health Organization Well-Being Index (0–100, higher scores indicate better well-being); SF-12 MCS, Mental Component Summary (0–100, higher scores indicate better mental health); SF-12 PCS, Physical Component Summary (0–100, higher scores indicate better physical health).

Regarding physical health outcomes, higher baseline ILOC was associated with better physical health-related quality of life (SF-12 PCS) at 4 months (β = 0.04, p = 0.022), but this effect was not sustained at 12 months. No significant associations were found between baseline ILOC and changes in sedentary time, blood pressure, or waist circumference (S2 Table).

### Change in ILOC

**Fig 2:**
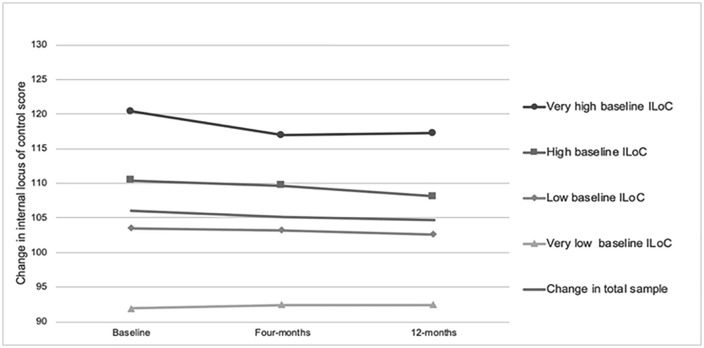
Change in internal locus of control (Duttweiler score) for very low baseline, low baseline, high baseline and very high baseline ILOC groups among baseline, 4-months and 12-months.

Overall, the total sample showed no significant net change in ILOC scores at 4 months (mean change: + 0.07, 95% CI [−0.63, + 0.77]) or 12 months (mean change: −0.38, 95% CI [−0.86, + 0.10]). However, stratified analysis revealed a clear pattern consistent with regression to the mean: participants with “Very High” baseline LOC experienced significant reductions at both time points, while those with “Very Low” baseline LOC showed significant increases (Fig 2 and S4 Table).

## Discussion

Participants with higher ILOC at baseline were, on average, older, more likely to have previously participated in the program, and more likely to report health-related motivations. Consistent with our hypotheses, higher baseline ILOC was positively associated with program engagement. It was associated with significantly higher average daily step counts and a greater likelihood of meeting the 10,000-step daily goal. However, this higher step engagement did not translate into a significant association with meeting WHO physical activity guidelines at four months after full adjustment, a discrepancy we discuss below. Previous evidence has reported that employees with higher ILOC are more likely to participate in health promotion programs and in moderate-to-vigorous physical activity, and our findings partially support this, with a clear positive association observed for pedometer-based step outcomes but not for self-reported guideline adherence [31]. It is worth noting, however, that higher step counts did not uniformly translate into meeting formal physical activity guidelines. This discrepancy likely reflects a measurement distinction: pedometers capture total ambulatory movement throughout the day, including low-intensity incidental walking, whereas physical activity guidelines are based on sustained bouts of at least moderate intensity. Consequently, participants may have substantially increased their overall daily movement – as reflected in higher step counts – without necessarily accumulating sufficient moderate-to-vigorous physical activity to meet self-reported guideline adherence [32].

Mental health outcomes showed time-dependent associations with ILOC. Higher ILOC was associated with better overall mental health-related quality of life at both 4 and 12 months. However, at the 4-month mark, higher ILOC was also associated with increased psychological distress. This short-term distress dissipated by 12 months, suggesting an adaptation process. This may reflect that individuals with high ILOC believe outcomes depend on their own actions [8]. One possible explanation is that, in the context of a structured intervention with daily targets, this belief likely enhances active engagement, which supports positive self-esteem and general mental health. However, this same sense of responsibility may induce performance anxiety when striving to meet daily goals, especially amidst competing workplace demands [33,34]. Additionally, the competitive and team-based structure of the GCC® may have amplified these effects through social comparison processes. Social comparison theory suggests that individuals evaluate their abilities and performance by comparing themselves with relevant others, which may generate negative affect and reduced self-esteem when perceived performance falls short of peers [35,36]. In the GCC®, participants could directly compare their team’s progress against other teams on a virtual leaderboard, creating a context in which upward social comparison was highly salient. For individuals with high ILOC, who tend to assume greater personal responsibility for outcomes, unfavourable team rankings may have been internalised as personal failures rather than attributed to situational factors, thereby amplifying self-blame and contributing to the increased psychological distress observed at four months. K10 psychological distress scores returned to baseline by 12 months, suggesting that early-intervention-related stress diminished over time. This allowed acute distress to subside while improvements in mental health persisted. This highlights the need for workplace programs to support high-ILOC employees in managing the immediate pressure of goal attainment to prevent burnout.

While the total sample showed no significant net change in LOC scores, stratified analysis revealed a clear pattern of regression to the mean, with participants in the highest baseline ILOC quartile showing significant declines and those in the lowest quartile showing significant increases. Such individuals may have initially overestimated their personal control, leading to higher baseline scores; as the program progressed, these perceptions may have been tempered by practical constraints such as work demands and limited time, resulting in a more realistic self-assessment [37]. Participants may also have experienced intervention fatigue as the initial novelty of the program wore off, contributing to lower reported LOC at follow-up. The Duttweiler Internal Control Index assesses LOC along a continuum from less to more internally focused, rather than contrasting internal and external control as separate dimensions [25,38].

The main limitation of this study is the lack of a control group, which means that a cause-and-effect relationship between ILOC and changes in outcomes cannot be established. Additionally, separate regression models were used at each time point rather than longitudinal models, as the primary exposure was time-invariant. Second, the analyses were based on complete-case data from participants who provided LOC and outcome measures at all time points. Our attrition analysis indicated that retained participants were significantly older than those who dropped out. While we adjusted for age in all regression models to mitigate this, the findings may be more applicable to older employees who are generally more likely to remain in workplace programs. Third, the study was undertaken during the colder winter months, when people are typically less active; participants might have demonstrated greater program benefits if the evaluation had been repeated in warmer months [39]. Fourth, interventions and research studies often attract participants with relatively positive health behaviors, known as the healthy cohort effect [40]. Fifth, the use of pedometers reflects the technology available at the time and may limit comparability with more recent accelerometer- or smartphone-based monitoring [32]. Participants may therefore have increased walking without consistently engaging in guideline-level moderate-intensity activity. Finally, the data were collected in 2008–09 but have been analyzed through a present-day lens. In 2007–08, around 62% of adults did not meet recommended physical activity guidelines compared with more recent estimates of approximately 31% in 2023–24 [41]. Over the same period, there has been a decline in manual labor occupations [42] and an increase in digital entertainment, meaning that many adults continue to engage in highly sedentary behaviors [43]. We therefore consider our findings to remain relevant for modern sedentary workplaces, while recognizing that the technological and policy context for physical activity promotion has evolved.

The strengths of the study include the relatively large sample size, longitudinal design, and detailed psychosocial and behavioral measures, as well as its focus on tertiary-educated adults employed in sedentary occupations. The GCC® Evaluation Study was conducted before the widespread adoption of accelerometers, smartphones, and gamified fitness platforms, providing a useful pre-digital benchmark for how psychological factors such as ILOC influence engagement in workplace activity programs. Our findings, together with prior outcomes from the GCC® Evaluation Study, help to fill a gap in the literature exploring pedometer-based workplace programs and locus of control.

In conclusion, ILOC is significantly associated with engagement in a workplace physical activity intervention, with higher ILOC associated with greater step-count performance and step-goal attainment. However, the potential for short-term distress among highly engaged employees suggests a need for balanced intervention designs. Future workplace interventions could benefit from integrating ILOC-sensitive design principles. For instance, employees with lower ILOC may require structured scaffolding like automated reminders, whereas high-ILOC individuals might thrive with greater autonomy balanced by non-competitive feedback to mitigate performance anxiety. Such tailored approaches are key to supporting sustainable behavior change and employee wellbeing.

## Supporting information

S1 Table Comparison of baseline characteristics between participants included in the final analysis and those excluded or lost to follow-up. (DOCX)

S2 Table Adjusted four-month and 12-month changes in secondary sedentary and anthropometric outcomes. (DOCX)

S3 Table Sensitivity analysis of the change in average steps per unit increase in LOC. Model 1: unadjusted; Model 2: adjusted for gender, age, and BMI; Model 3: adjusted for gender, age, BMI, physical activity, sitting time, well-being factors, health-related quality of life, systolic and diastolic blood pressure, and waist circumference.
(DOCX)

S4 Table Immediate and long-term change in internal locus of control (Duttweiler score) associated with participation in a physical activity workplace program (Duttweiler completed at all three timepoints). (DOCX)

S5 Table Covariables and their types. (DOCX)
