# Supplementary material for: Higher internal locus of control is associated with higher performance in a workplace walking intervention, Global Corporate Challenge®
Source: PLoS One. 2026 Jun 1;21(6):e0349934. doi: 10.1371/journal.pone.0349934 (PMC13225370; doi:10.1371/journal.pone.0349934)
Supplement: S1 Table — (DOCX) [file pone.0349934.s001.docx]

**Supplementary Materials**

**Supplementary table 1.** Comparison of baseline characteristics between participants included in the final analysis and those excluded or lost to follow-up.

| **Characteristic** | **Included in Analysis (n=426)** | **Excluded/Lost to Follow-up (n=385)** | **P-value** |
| --- | --- | --- | --- |
| **Demographics** |  |  |  |
| Age (years), mean (SD) | 41.3 (10.2) | 37.9 (10.5) | <0.001* |
| Female sex, n (%) | 246 (57.7%) | 236 (61.3%) | 0.304 |
| Tertiary education, n (%)ᵃ | 344 (80.8%) | 251 (78.2%) | 0.390 |
| **Behavioral factors** |  |  |  |
| Prior GCC participation, n (%)ᵇ | 98 (23.0%) | 62 (20.3%) | 0.376 |
| Meeting PA Guidelines (baseline), n (%) | 177 (41.6%) | 104 (34.3%) | 0.048* |
| **Health** |  |  |  |
| BMI (kg/m²), mean (SD) | 26.8 (4.8) | 26.5 (5.0) | 0.389 |
| **Psychological measures** |  |  |  |
| Baseline LOC score, mean (SD) | 106.1 (11.2) | 105.8 (11.0) | 0.658 |
| Baseline Well-being (WHO-5), mean (SD) | 60.1 (18.9) | 59.9 (19.4) | 0.886 |

Data are presented as Mean (Standard Deviation) for continuous variables and n (%) for categorical variables. P-values were calculated using independent t-tests for continuous variables and Chi-square tests for categorical variables. Abbreviations: SD, Standard Deviation; PA, Physical Activity; BMI, Body Mass Index; LOC, Locus of Control; WHO-5, World Health Organization Well-Being Index. * Indicates statistical significance (p < 0.05). ᵃ Includes university or vocational training. ᵇ Global Corporate Challenge.
