# Supplementary material for: Higher internal locus of control is associated with higher performance in a workplace walking intervention, Global Corporate Challenge®
Source: PLoS One. 2026 Jun 1;21(6):e0349934. doi: 10.1371/journal.pone.0349934 (PMC13225370; doi:10.1371/journal.pone.0349934)
Supplement: S2 Table — (DOCX) [file pone.0349934.s002.docx]

**Supplementary table 2.** Adjusted four-month and 12-month changes in secondary sedentary and anthropometric outcomes.

| **Measure** | **Four-month change (95% CI)** | **p-value** | **12-month change (95% CI)** | **p-value** |
| --- | --- | --- | --- | --- |
| **Sitting time (hrs per day)** | | | | |
| Weekday | -0.002 (-0.051, 0.047) | 0.9274 | -0.003 (-0.049, 0.042) | 0.8809 |
| Weekend | -0.003 (-0.019, 0.013) | 0.6462 | -0.003 (-0.021, 0.015) | 0.7174 |
| **Anthropometric measures** |  |  |  |  |
| Systolic blood pressure (mmHg) | -0.001 (-0.123, 0.120) | 0.9807 | -0.003 (-0.074, 0.069) | 0.9304 |
| Diastolic blood pressure (mmHg) | -0.018 (-0.115, 0.080) | 0.6936 | -0.031 (-0.100, 0.038) | 0.3365 |
| Waist circumference | -0.004 (-0.048, 0.039) | 0.8218 | -0.020 (-0.080, 0.040) | 0.4689 |

Values represent adjusted regression coefficients (95% Confidence Intervals) derived from linear regression models adjusted for baseline values and covariates. Abbreviations: CI, Confidence Interval.
