# Supplementary material for: Higher internal locus of control is associated with higher performance in a workplace walking intervention, Global Corporate Challenge®
Source: PLoS One. 2026 Jun 1;21(6):e0349934. doi: 10.1371/journal.pone.0349934 (PMC13225370; doi:10.1371/journal.pone.0349934)
Supplement: S3 Table — Model 1: unadjusted; Model 2: adjusted for gender, age, and BMI; Model 3: adjusted for gender, age, BMI, physical activity, sitting time, well-being factors, health-related quality of life, systolic and diastolic blood pressure, and waist circumference. (DOCX) [file pone.0349934.s003.docx]

**Supplementary Table 3.** Sensitivity analysis of the change in average steps per unit increase in LOC. Model 1: unadjusted; Model 2: adjusted for gender, age, and BMI; Model 3: adjusted for gender, age, BMI, physical activity, sitting time, well-being factors, health-related quality of life, systolic and diastolic blood pressure, and waist circumference.

| **Model** | **Adjusted Covariates** | **LOC Coefficient (95% CI)** | **p-value** | **R²** | **Adj R²** |
| --- | --- | --- | --- | --- | --- |
| Model 1 | None | 52.95 (21.50, 84.39) | 0.001 | 0.025 | 0.023 |
| Model 2 | Gender, Age, BMI | 52.32 (20.16, 84.47) | 0.001 | 0.05 | 0.041 |
| Model 3 | Gender, age, BMI, physical activity, sitting times, well-being factors, health related quality of life, systolic blood pressure, diastolic blood pressure and waist circumstance | 47.96 (12.86, 83.06) | 0.008 | 0.167 | 0.138 |
