# Supplementary material for: Higher internal locus of control is associated with higher performance in a workplace walking intervention, Global Corporate Challenge®
Source: PLoS One. 2026 Jun 1;21(6):e0349934. doi: 10.1371/journal.pone.0349934 (PMC13225370; doi:10.1371/journal.pone.0349934)
Supplement: S4 Table — (DOCX) [file pone.0349934.s004.docx]

**Supplementary Table 4.** Immediate and long-term change in internal locus of control (Duttweiler score) associated with participation in a physical activity workplace program (Duttweiler completed at all three timepoints).

|  | Baseline | Four-month | 12-month | Baseline to four-months | | Baseline to 12-months | |
| --- | --- | --- | --- | --- | --- | --- | --- |
| **Change in ILOC** | **(Mean±SD)** | **(Mean±SD)** | **(Mean±SD)** | **Mean (95% CI)** | **p-value** | **Mean (95% CI)** | **p-value** |
| **Total sample (n = 426)** | 106.0±11.1 | 104.8±11.9 | 104.5±12.4 | -1.0 (-1.6, -0.3) | 0.003 | -1.6 (-2.3, -0.9) | <0.001 |
| **Very low baseline ILoC (n = 113)** | 91.6±5.9 | 92.3±9.3 | 92.4±8.1 | +0.6 (-0.7, 1.9) | 0.352 | +0.7 (-0.5, 1.9) | 0.229 |
| **Low baseline ILoC (n = 110)** | 103.5±2.6 | 103.0±7.5 | 102.3±7.9 | -0.5 (-1.8, 0.7) | 0.41 | -1.2 (-2.5, 0.2) | 0.084 |
| **High baseline ILoC (n = 100)** | 110.2±1.6 | 109.3±6.7 | 107.4±8.8 | -1.1 (-2.3, 0.2) | 0.087 | -2.9 (-4.4, -1.4) | <0.001 |
| **Very high baseline ILoC (n = 103)** | 120.1±5.0 | 116.9±7.4 | 117.2±9.7 | -3.1 (-4.2, -2.0) | <0.001 | -3.5 (-5.0, -2.0) | <0.001 |
