# Supplementary material for: Higher internal locus of control is associated with higher performance in a workplace walking intervention, Global Corporate Challenge®
Source: PLoS One. 2026 Jun 1;21(6):e0349934. doi: 10.1371/journal.pone.0349934 (PMC13225370; doi:10.1371/journal.pone.0349934)
Supplement: S5 Table — (DOCX) [file pone.0349934.s005.docx]

**Supplementary Table 5. Covariables and their types**

| **Demographics** |  |  |
| --- | --- | --- |
| Age (years) | Continuous | Mean ± SD |
| Gender (Male/Female)a | Binary | Male / Female |
| Completion of Tertiary Education | Binary | Yes / No |
| Partner Status | Categorical | - Married or de facto |
|  |  | - Widowed, separated, or divorced |
|  |  | - Never married |
| Socioeconomic Status (SEIFA) | Categorical | - Most Advantaged |
|  |  | - Advantaged |
|  |  | - Disadvantaged |
|  |  | - Most Disadvantaged |
| Occupation | Categorical | - Professional |
|  |  | - Associate professional |
|  |  | - Manager |
|  |  | - Clerical or Service |
|  |  |  |
| **GCC Intervention Measures** |  |  |
| Prior GCC® Participation | Binary | Yes / No |
| Motivation for Participation (each item)b | Binary (per item) | - Health (Yes/No) |
|  |  | - To look my best (Yes/No) |
|  |  | - Fitness (Yes/No) |
|  |  | - Colleagues (Yes/No) |
|  |  | - Friends/family (Yes/No) |
|  |  |  |
| **Behavioural Measures** |  |  |
| Fruit Intake | Binary | Meeting guidelines (Yes) / Not meeting guidelines (No) |
| Vegetable Intake | Binary | Meeting guidelines (Yes) / Not meeting guidelines (No) |
| Alcohol Consumption | Binary | Meeting guidelines (Yes) / Not meeting guidelines (No) |
| Smoking Status | Binary | Smoker / Non-smoker |
| Physical Activity | Binary | Meeting PA guidelines (Yes) / Not meeting (No) |
| Sitting Time (Weekday & Weekend) | Continuous | Hours per day (mean ± SD) |
| Takeaway Dinner Frequency | Categorical | - Once or less per month |
|  |  | - About once a week |
|  |  | - More than once a week |
|  |  |  |
| **Psychosocial Measures** |  |  |
| WHO-5 Well-being (score) | Continuous | Mean ± SD (0–100 scale) |
| WHO-5 Well-being (positive category) | Binary | Yes / No |
| SF-12 Mental Health Component | Continuous | Mean ± SD (0–100 scale) |
| SF-12 Physical Health Component | Continuous | Mean ± SD (0–100 scale) |
| K10 Scores | Categorical | - Low |
|  |  | - Moderate |
|  |  | - High |
|  |  | - Very High |
|  |  |  |
| **Anthropometric Measures** |  |  |
| Systolic Blood Pressure (mmHg) | Continuous | Mean ± SD |
| Diastolic Blood Pressure (mmHg) | Continuous | Mean ± SD |
| Heart Rate (beats per minute) | Continuous | Mean ± SD |
| Weight (kg) | Continuous | Mean ± SD |
| Body Mass Index (kg/m²) | Continuous | Mean ± SD |
| Waist Circumference (cm) | Continuous | Mean ± SD |
